# Affinity proteomic profiling of plasma for proteins associated to area-based mammographic breast density

**DOI:** 10.1186/s13058-018-0940-z

**Published:** 2018-02-14

**Authors:** Sanna Byström, Martin Eklund, Mun-Gwan Hong, Claudia Fredolini, Mikael Eriksson, Kamila Czene, Per Hall, Jochen M. Schwenk, Marike Gabrielson

**Affiliations:** 1 Science for Life Laboratory, School of Biotechnology, KTH – Royal Institute of Technology, Stockholm, Sweden; 2 Department of Medical Epidemiology and Biostatistics, Karolinska Institutet, Nobels väg 12A, -171 77 Stockholm, SE Sweden; 3 Department of Oncology, South General Hospital, Stockholm, Sweden

**Keywords:** Mammographic breast density, Plasma, Protein profiling, Suspension bead array, Affinity proteomics, KARMA cohort

## Abstract

**Background:**

Mammographic breast density is one of the strongest risk factors for breast cancer, but molecular understanding of how breast density relates to cancer risk is less complete. Studies of proteins in blood plasma, possibly associated with mammographic density, are well-suited as these allow large-scale analyses and might shed light on the association between breast cancer and breast density.

**Methods:**

Plasma samples from 1329 women in the Swedish KARMA project, without prior history of breast cancer, were profiled with antibody suspension bead array (SBA) assays. Two sample sets comprising 729 and 600 women were screened by two different SBAs targeting a total number of 357 proteins. Protein targets were selected through searching the literature, for either being related to breast cancer or for being linked to the extracellular matrix. Association between proteins and absolute area-based breast density (AD) was assessed by quantile regression, adjusting for age and body mass index (BMI).

**Results:**

Plasma profiling revealed linear association between 20 proteins and AD, concordant in the two sets of samples (*p* < 0.05). Plasma levels of seven proteins were positively associated and 13 proteins negatively associated with AD. For eleven of these proteins evidence for gene expression in breast tissue existed. Among these, ABCC11, TNFRSF10D, F11R and ERRF were positively associated with AD, and SHC1, CFLAR, ACOX2, ITGB6, RASSF1, FANCD2 and IRX5 were negatively associated with AD.

**Conclusions:**

Screening proteins in plasma indicates associations between breast density and processes of tissue homeostasis, DNA repair, cancer development and/or progression in breast cancer. Further validation and follow-up studies of the shortlisted protein candidates in independent cohorts will be needed to infer their role in breast density and its progression in premenopausal and postmenopausal women.

**Electronic supplementary material:**

The online version of this article (10.1186/s13058-018-0940-z) contains supplementary material, which is available to authorized users.

## Background

Mammographic breast density is one of the strongest risk factors for breast cancer. Women with high breast density have 4–6-fold increased risk of breast cancer as compared to women with low breast density [1–4]. Reflecting the composition of fibroglandular and fat tissue in the breast, mammographic breast density is inversely related to age and higher body mass index (BMI). Radiologically dense tissue, such as stromal and epithelial tissue, appears white on a mammogram, whereas the radiologically lucent fat tissue appears dark [5]. Several breast cancer risk factors are known to influence breast density [6]. It has been shown that body weight and reproductive and lifestyle factors explain an estimated 20–30% of the difference in density between women [7]. Through twin studies, we and others have estimated the heritability of percent density to be around 65% [7–9].

Despite the strong and independent association between mammographic breast density and breast cancer risk, little is known about the biological mechanisms behind this risk factor. Identifying determinants of density may provide insights into the aetiology of breast cancer. It may also be useful for better identifying women at increased risk of developing breast cancer.

Considerable effort has been made to identify biomarkers for early detection and/or monitoring of breast cancer. Although a few potential plasma protein targets have been identified [10], validation and reproducibility have thus far not been satisfactory for clinical implementation. Prior investigations of plasma markers associated with breast density have mainly focused on endogenous hormones and inflammatory markers with inconsistent or negative results [6]. No putative independent markers of mammographic density have so far been identified after adjustment for BMI and other confounding factors.

Blood plasma is well-suited for expanded affinity proteomic analysis as it enables a direct but less invasive view into the health status compared to biopsy sampling. Affinity proteomics assays using antibodies with suspension bead arrays (SBA) have been utilised for plasma protein profiling within the context of various diseases including cancer [11]. The approach allows for many proteins to be screened in small plasma volumes of a large number of samples [12], thus enabling large-scale proteomic investigations of body fluids like plasma.

In this study, we used a multiplexed affinity proteomics assay with antibodies from the Human Protein Atlas (HPA) [13] to screen proteins in plasma of women without any prior history of breast cancer, and who were enrolled in a unique prospective population-based cohort in Sweden, the Karolinska mammography project for risk prediction for breast cancer (KARMA) cohort [14, 15]. The aim of this exploratory approach was to identify density-associated proteins, to improve our still limited understanding of mammographic breast density as a risk factor for breast cancer.

## Methods

### Study populations and data collection

This study included samples collected from participants of the KARMA cohort [14]. KARMA is a population-based cohort initiated in January 2011, which comprises 70,877 women attending routine mammography screening or clinical mammography at four hospitals in Sweden [14, 15]. The overarching goal of KARMA is to reduce the incidence and mortality of breast cancer by focusing on individualised prevention and screening.

Raw (unprocessed) digital mammograms for each study participant were collected at KARMA study enrolment [14, 15]. Mammograms were taken from cranial-caudal and mediolateral oblique views by full-field digital mammography. Mammographic density was measured two-dimensionally as an absolute dense area (AD) (cm^2^) using the newly developed in-house STRATUS program as previously described [15, 16] and three-dimensionally as an absolute dense volume (VD) (cm^3^) using the automated Volpara system. STRATUS analyses both raw and processed mammograms and estimates the breast and dense area based on mammographic textures. Each pattern segment is analysed for several statistical features including pattern area, circumference, intensity, positioning, relation to other areas and shape. This quantified texture structure of the breast is compared to a reference library of matching breast texture-density-level pairs. The reference library was created using the penalised lasso regression machine-learning method.

The total mammographic dense area and percent mammographic density did not differ significantly between the two sample sets (*p* = 0.80 and *p* = 0.90, respectively). AD and VD measures from the right breast were considered for statistical analysis.

**Fig. 1:**
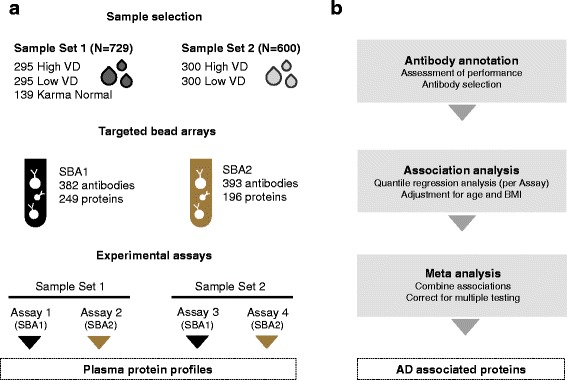
Study overview. **a** Samples comprised plasma from women with high and low absolute volumetric breast density (High VD and Low VD) matched on age and body mass index (BMI) from the population-based KARMA cohort (Sample Set 1, *N* = 729; Sample Set 2, *N* = 600). In Sample Set 1, an additional set of 139 individuals (Karma Normal) was included. For the experimental procedure, two antibody suspension bead arrays (SBA1 and SBA2) were created with antibodies available from the Human Protein Atlas: 249 and 196 proteins were targeted. These proteins were selected from breast-cancer-related literature and proteins annotated to extracellular matrix. Both bead arrays were used for the screening of each plasma sample set (Assay 1–4). **b** The plasma protein profiles that were generated in the four assays were annotated and filtered based on technical quality assessments. Association with absolute area-based breast density (AD) was then assessed by quantile regression analysis, adjusting for age and BMI. Combining the results from regression analyses performed within each sample set by meta-analysis resulted in candidate protein profiles with linear associations to AD

KARMA participants were included in the study based on measured VD and selected from the total KARMA study population (*N* = 70,773). For practical reasons, the study was conducted in two phases resulting in two sample sets; sample set 1 included 729 women from three sample groups and sample set 2 included 600 women from two sample groups (Table 1 and Fig. 1). No participant had a prior history of breast cancer or other malignant cancer at the time of sampling. One individual developed breast cancer 2 years after blood draw.

**Table 1:** Sample demographics

	Sample set 1				Sample set 2		
Characteristics	All samples *N* = 729	Low mammographic density *N* = 295	High mammographic density *N* = 295	Karma normal *N* = 139	All samples *N* = 600	Low mammographic density *N* = 300	High mammographic density *N* = 300
Mean (SD)							
Age, years	53.6 (9.5)	52.9 (9.5)	52.9 (9.5)	56.5 (9.1)	54.2 (9.6)	54.2 (9.6)	54.2 (9.6)
BMI, kg/m^2^	24.4 (3.1)	24.4 (2.6)	24.2 (2.6)	25.2 (4.5)	24.1 (2.7)	24.2 (2.7)	24.1 (2.7)
Absolute breast dense area, cm^2^	35.4 (29.3)	17.0 (13.9)	56.6 (30.0)	29.4 (22.1)	37.3 (31.2)	15.7 (12.5)	58.9 (29.4)
Absolute volumetric breast density, cm^3^	72.0 (43.6)	33.9 (10.0)	108.5 (36.2)	75.3 (32.4)	73.0 (49.5)	33.4 (10.8)	112.5 (40.7)
Age at menarche, years	13.1 (1.5)	13.0 (1.5)	13.2 (1.5)	13.1 (1.4)	13.1 (1.4)	13.1 (1.4)	13.1 (1.3)
Age at first birth, years	27.5 (5.4)	27.5 (5.3)	28.2 (5.7)	26.2 (4.8)	27.8 (5.2)	27.4 (5.1)	28.3 (5.3)
Age at menopause, years	50.2 (5.5)	50.3 (5.8)	50.0 (5.1)	50.3 (5.6)	49.6 (5.3)	49.2 (5.4)	50.1 (5.2)
Number (percent)							
Nulliparous	86 (11.9)	18 (6.1)	51 (17.3)	17 (12.7)	103 (17.2)	37 (12.3)	66 (22.0)
Menopausal status							
Premenopausal	337 (46.4)	140 (47.5)	157 (53.2)	40 (29.4)	268 (44.7)	129 (43.0)	139 (46.3)
Postmenopausal	389 (53.6)	155 (52.5)	138 (46.8)	96 (70.6)	332 (55.3)	171 (57.0)	161 (53.7)
HRT use ever							
No	527 (79.0)	233 (79.0)	243 (82.4)	96 (71.6)	488 (81.3)	241 (80.3)	247 (82.39
Yes	152 (21.0)	62 (21.0)	52 (17.6)	38 (28.4)	112 (18.7)	59 (19.7)	53 (17.7)

Number of individuals with missing data for the following variables: body mass index (BMI) (*N* = 1), mammographic density (N = 1), age at menarche (*N* = 31), age at first birth (*N* = 198), age at menopause (*N* = 420), parity (*N* = 5), postmenopausal status (*N* = 3), and hormone replacement therapy (HRT) use ever (*N* = 5)

*SD* standard deviation

**Fig. 2:**
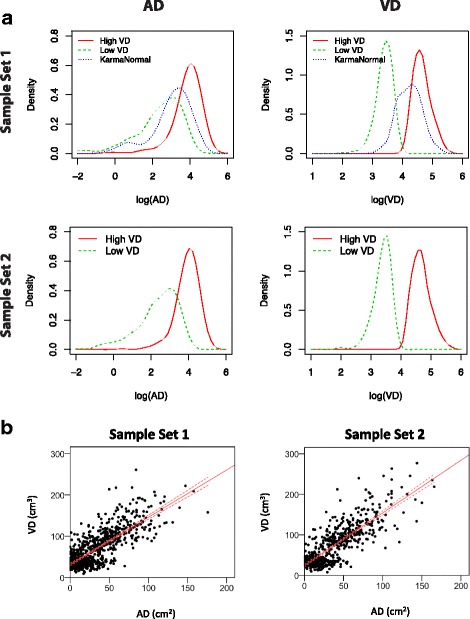
Mammographic breast density within sample groups. **a** Density plots show the distribution of absolute area-based breast density (AD) (cm^2^) and absolute volumetric breast density (VD) (cm^3^) within the sample groups representing the original sample selection (Sample Set 1, High VD, Low VD and KarmaNormal; Sample Set 2, High VD and Low VD). Mean values of AD and VD in all sample groups from both sample sets can be found in Table 1. **b** Correlation between AD and VD measurements within Sample Set 1 (*rho* = 0.71) and Sample Set 2 (*rho* = 0.75)

For each sample set, women were allocated into two subsets of VD (high and low). The high-density sample groups (sample set 1, median VD = 104.9 cm^3^; sample set 2, VD = 100.2 cm^3^) were women from the highest quintile of absolute volumetric density in the KARMA cohort. The low-density sample groups (sample set 1, median VD = 33.5 cm^3^; sample set 2, median VD = 33.5 cm^3^) were women from the lowest quintile of absolute volumetric density in KARMA. The sample groups (high and low VD) were matched on age and BMI (Fig. 2). An additional 139 samples from another KARMA study (denoted “Karma Normal”) were selected in the same way based on the highest and lowest quintiles of absolute volumetric density (median VD = 68.3 cm^3^) and included in sample set 1 (Fig. 2). Karma Normal is a nested study within KARMA with the objective to study normal breast physiology and only includes samples from healthy participants in KARMA, without any history of breast cancer or other cancers. Karma Normal has been described in detail elsewhere [17]. Participants in both sample sets were matched to the Information Network for Cancer treatment (INCA) to ensure disease-free status at the time of sample collection. BMI was calculated at the time of the mammogram and was based on self-reported height and weight. Distributions of sample characteristics and breast cancer risk factors were similar between the two study sample sets (Table 1) and between each study sample set and the total KARMA cohort. Each study participant signed an informed consent form before joining the KARMA project. The Stockholm ethical review board approved the study (2010/958-31/1).

### Sample collection

Non-fasting EDTA plasma samples of peripheral blood were collected from the KARMA study participants at enrolment [14, 15]. All blood samples were handled in accordance to a strict 30-h cold-chain protocol and were processed in the Karolinska Institutet high-throughput biobank. The majority (97.5%) of blood samples were taken on the same day as the mammogram. Mean time between the mammogram and blood sample collection was 4.8 h (SD 57.6 h). The time interval from the mammogram to blood collection did not differ significantly between sample set 1 and sample set 2 (*p* = 0.80).

### Target and antibody selection

For multiplexed protein profiling, sets of 382 and 393 antibodies derived from the Human Protein Atlas [13] were used. These targeted a total 445 unique protein-encoding genes, and a complete list of all antibodies included in the study is provided in Additional file 1: Tables S1-S2. The 382 antibodies included in the first suspension bead array (SBA1) were selected based on a possible relationship with mammographic breast density, cancer development and/or progression or tissue composition and/or remodelling. The 393 antibodies included in the second bead array (SBA2) targeted proteins annotated to extracellular matrix (Uniprot.org; *N* = 156) [18] and proteins enriched in breast tissue according to RNA sequencing (RNAseq) data [13]. The list also included antibodies selected from immunohistochemistry (IHC) primary data [13]. Further details about antibody generation and selection can be found in Additional file 2.

### Antibody bead array assays

Antibody bead arrays were generated using carboxylated magnetic beads of up to 393 unique bead identities (MagPlex-C, Luminex Corp.) as previously described [12]. All plasma samples within each study set were retrieved from the biobank and analysed at the same time points. Plasma samples stored at − 80 °C were thawed at 4 °C and transferred to 96-well microtitre plates in a semi-randomised plate layout, where samples from different sampling locations were balanced across the different plates and each matched pair of the two sample groups (high and low VD) were placed within the same plate. The randomised plate layouts resulted in an even distribution of AD across all 96-well plates (Kruskal-Wallis *p* values 0.94 and 0.57 for sample sets 1 and 2, respectively). All plates included four aliquot replicates from a crude plasma pool from all individuals included in that study set. Samples were biotinylated, diluted, heat-treated at 56 °C and combined with the bead array on two separate 384-well assay plates in accordance with previously described protocols [19]. Further details can be found in Additional file 2.

### Methods for antibody validation

Different types of assays were used to validate the antibodies. Detailed descriptions about epitope mapping by high-density peptide arrays, western blot and immuno-capture mass spectrometry analysis of plasma samples can be found in Additional files 1 and 2**.**

### Data processing and quality control

Data from SBA assays were processed separately according to the following procedure: blank (sample-free with buffer) wells were excluded from analysis. In sample set 2, the replicated data from one 96-well plate was used only for quality control; meaning only one of each sample in a duplicated pair was included for statistical analysis. Outlying samples, detected by robust principal component analysis (PCA) [20], were replaced by missing values (N/A) using the “rrcov” R package. Probabilistic quotient normalisation [21] was then applied for all data points originating from each 96-well plate, followed by between-plate normalisation using a multidimensional normalisation method [22]. Prior to statistical analyses, antibody profiles were annotated based on assay performance. The annotations were based on four different criteria, including median signal intensities above that of the negative control bead identity (rIgG). A more detailed description is provided in Additional file 2. Filtering of antibody profiles based on such technical quality assessment resulted in a refined list of 245 (SBA1) and 244 (SBA2) antibodies against a total number of 357 proteins that were targeted within each study set. PCA was applied for quality control and to detect potential sampling location effects. Prior to PCA, data were log-transformed, centred and subjected to unit variance scaling, and missing data points were replaced by the median of the complete data set.

### Experimental study design

The initial aim of the study was to contrast plasma protein profiles of women with high and low VD. However, during the proceeding time, a study by Nguyen et al. showed that breast cancer risk is more strongly associated with the denser part of the breast [23]. AD is thus likely a better representation of the true dense tissue in the breast. We therefore updated our strategy and performed our density-protein association analyses using absolute area-based density measures, which targets the most radio-dense tissue in the breast. Identification of protein profiles in relation to AD while controlling for age and BMI is thus relevant for providing new biological insights into the mechanisms of mammographic breast density. Accordingly, and prior to data analyses, we decided to use AD as our primary endpoint. We also report results according to our original design, using VD as endpoints and samples matched for age and BMI (Fig. 4 and Additional file 2).

### Statistical analysis

For contrasting high and low VD, the paired Wilcoxon signed-rank test of normalised and log transformed data was used. In order to keep the matching of age and BMI between paired sample IDs, sample IDs matched to those classified as outliers, based on signal intensities in robust PCA in the experimental data, were removed prior to analysis. This resulted in 5 matched pairs (10 samples) within sample set 1 and 6 matched pairs (12 samples) within sample set 2 being excluded from two-group comparisons.

To assess the association between antibody profiles and AD, normalised, log-transformed data scaled to unit variance were used for statistical analysis. Eleven sample outliers, as identified by robust PCA, were excluded from the statistical analysis. Quantile regression models were computed using the “quantreg” package in R. In both sample sets, the correlation between AD and BMI differed between the matched groups of high and low VD; there was stronger negative correlation between AD and BMI in the low VD group (*rho* = −0.51 and −0.53, respectively) than in the high VD group (*rho* = −0.19 and −0.12, respectively) (see Additional file 2: Figure S1). Consequently, the effect of AD was adjusted for age, BMI, sample group (high or low VD) and the interaction between BMI and VD. When stated, *p* values from each study set (set 1 and set 2) were combined by Fisher’s method and adjusted for multiple testing using the Benjamini-Hochberg method (referred to as “adj. *p*”). Data analysis and statistical analysis were performed in R.

## Results

### Plasma profiles in relation to age and BMI

First, we investigated the associations between the plasma profiles and age and BMI. Both variables influence AD but were not associated with one another in the studied sample sets (*p* > 0.1). Age was associated with 11 plasma profiles at *p* < 10^−10^, with concordant trends in both sample sets (Additional file 2: Table S3). Among these, the profiles for AMBN, TMEM86A, MLH1, PTGR1 and SPNS1 were less strongly associated with BMI (*p* > 0.001), and all but SPNS1 decreased with age. For association with BMI, the overall significance levels were lower compared to those for age, and there were concordant trends for 10 profiles in both sample sets (*p* < 10^−5^; Additional file 2: Table S4). Among the profiles associated with BMI, only TPP1 and ENG profiles were less strongly associated with age (*p* > 0.001). Interestingly, the trends of the slopes for BMI and age only differed for TPP1.

### Plasma profiles associated with AD

**Fig. 3:**
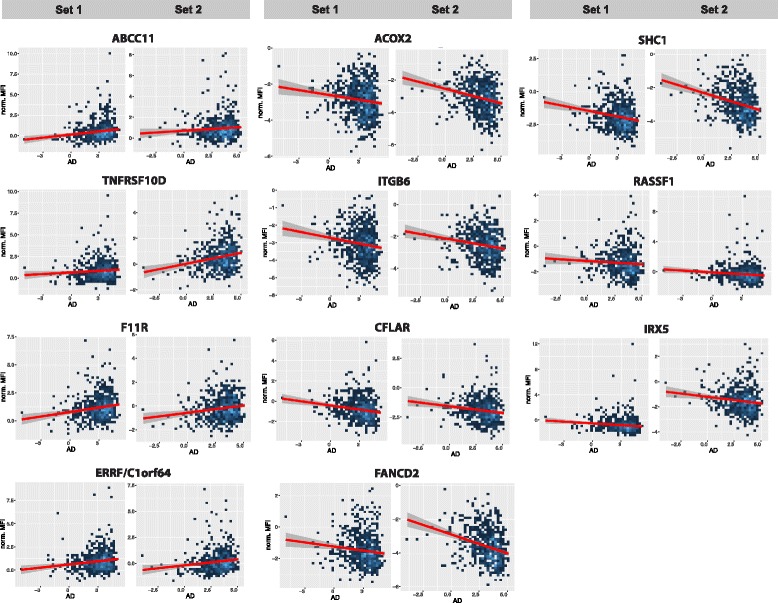
Associations between proteins and area-based mammographic breast density. The 11 candidate proteins expressed in breast tissue (see Tables 2 and 3) and their relationships with absolute area-based mammographic density (AD) are shown. Data from the analysis of both sample sets are shown. The red lines represent the linear relationship between the measured protein levels after adjusting for body mass index, absolute volumetric breast density (VD) and the interaction between AD and VD, stated as “norm. MFI”. The x-axis depicts the log-scaled distribution of AD values. The density of data points is shown on a coloured heatmap, where data points are binned into rectangles. Darker and lighter blue colours indicate lower and higher density of data points, respectively

We subsequently analysed the linear relation between protein profiles and AD. The data were adjusted for age, BMI and the interaction between BMI and VD group. The distributions of AD and VD within the three sample groups are illustrated in Fig. 2. Using quantile regression models, we identified 20 candidate profiles that were significantly associated with AD (*p* < 0.05) in both sample sets. All proteins remained significant (adj. *p* < 0.05) after combining the *p* values from both sample sets and adjusting for multiple testing. In total, 11 of the 20 proteins (55%) were negatively associated with AD (Tables 2 and 3). Among these were ACOX2, ITGB6 and SHC1, which had been observed as proteins strongly associated with age and BMI. Next, we investigated the candidates for expression in breast tissue. Annotations of gene expressions were obtained from publically available RNAseq or immunohistochemistry data (Additional file 2: Table S5) [13, 24]. Table 2 lists those 11 candidates for which gene or protein expression has been detected in human breast tissue. Figure 3 demonstrates linear associations with the eleven candidates and shows that plasma levels of ABCC11, TNFRSF10D, F11R and ERRF were positively associated with AD, while SHC1, CFLAR, ACOX2, ITGB6, RASSF1, FANCD2 and IRX5 were negatively associated with AD. The additional nine candidates lacking RNA expression in breast tissue are shown in Table 3.

**Table 2:** Protein profiles associated with area-based mammographic breast density in both study sets (*p* < 0.05)

					Sample set 1, *N* = 729		Sample set 2, *N* = 600		All samples	
Gene	Gene description	Annotated protein function^a^	HPA	ENSG	*P* value	Effect size	*P* value	Effect size	Trend	Adjusted *p* value^b^
ABCC11	ATP-binding cassette, sub-family C (CFTR/MRP), member 11	Membrane-associated transport protein	HPA031981	ENSG00000121270	0.002	0.08	0.00003	0.09	+	0.002
TNFRSF10D	Tumor necrosis factor receptor superfamily, member 10d, decoy with truncated death domain	Transmembrane signalling receptor regulating apoptosis	HPA065387	ENSG00000173530	0.002	0.16	0.03	0.08	+	0.002
F11R	F11 receptor	Epithelial cell-cell adhesion molecule.	HPA061700	ENSG00000158769	0.02	0.11	0.02	0.10	+	0.004
ERRF/C1orf64	ER-related factor (chromosome 1 open reading frame 64)	Steroid receptor associated and regulated protein	HPA026676	ENSG00000183888	0.007	0.13	0.002	0.11	+	0.007
ACOX2	Acyl-CoA oxidase 2, branched chain	Acyl-Coenzyme A oxidase involved in the degradation of long branched fatty acids	HPA064845	ENSG00000168306	0.003	−0.16	0.0001	−0.15	–	0.000005
ITGB6	Integrin, beta 6	Adhesion receptor signalling molecule	HPA023626	ENSG00000115221	0.000003	− 0.12	0.0009	− 0.15	–	0.00003
CFLAR	CASP8 and FADD-like apoptosis regulator	Cystein-type peptidase regulating apoptosis	HPA050009	ENSG00000003402	0.003	−0.12	0.003	−0.14	–	0.00009
FANCD2	Fanconi anaemia, complementation group D2	Complementation protein involved in homology-directed DNA repair	HPA054101	ENSG00000144554	0.0004	−0.24	0.02	−0.08	–	0.0001
SHC1	SHC (Src homology 2 domain containing) transforming protein 1	Adapter protein in signal transduction pathways	HPA001577	ENSG00000160691	0.0002	−0.20	0.008	−0.10	–	0.0002
RASSF1	Ras association (RalGDS/AF-6) domain family member 1	Signal transduction protein regulating apoptosis	HPA040735	ENSG00000068028	0.05	−0.10	0.02	−0.07	–	0.008
IRX5	Iroquois homeobox 5	Transcription factor involved in cell differentiation and cell cycle	HPA047130	ENSG00000176842	0.04	−0.10	0.02	−0.09	–	0.04

^a^Information extracted from the Human Protein Atlas [24]. More detailed descriptions of protein functions are given in “Discussion” and Additional file 2

^b^Fisher’s combined probability test, adjusted for multiple testing (Benjamini and Hochberg method)

**Table 3:** Candidate proteins without annotated gene expression in breast tissue

					Sample set 1, *N* = 729		Sample set 2, *N* = 600		All samples	
Gene	Gene description	Annotated protein function^a^	HPA	ENSG	*P* value	Effect size	*P* value	Effect size	Trend adjusted	Adjusted *p* value^b^
AGER	Advanced glycosylation end-product-specific receptor	Multi-ligand receptor member of immunoglobulin superfamily cell surface receptors	HPA069474	ENSG00000204305	0.0004	0.09	0.002	0.10	+	0.00001
CYP2S1	Cytochrome P450 family 2 subfamily S member 1	Monooxygenase superfamily of enzymes, unclear function in humans	HPA037692	ENSG00000167600	0.008	0.09	0.03	0.07	+	0.002
DEFB134	Defensin beta 134	Secreted antimicrobial protein	HPA044494	ENSG00000205882	0.03	0.13	0.008	0.07	+	0.03
CST6	Cystein E/M	Secreted proteinase inhibitor with protective and anti-metastatic functions	HPA044963	ENSG00000175315	0.02	−0.07	0.009	−0.09	–	0.002
DLX1	Distal-less homeobox 1	Member of homeobox transcription factor gene family	HPA007175	ENSG00000144355	0.02	−0.11	0.003	−0.14	–	0.02
F2	Coagulation factor II, thrombin	Part of first step of coagulation cascade.	HPA051476	ENSG00000180210	0.01	−0.17	0.01	−0.11	–	0.01
IL4	Interleukin 4	Pleiotropic cytokine produced by activated T cells	HPA042270	ENSG00000113520	0.002	−0.20	0.0001	−0.14	–	0.000003
LIN28B	Lin-28 homolog B	Member of lin-28 family of RNA binding proteins	HPA061745	ENSG00000187772	0.03	−0.08	0.01	−0.10	–	0.003
PSMA8	Proteasome subunit alpha 8	Peptide cleaving proteasome involved in histone	HPA049377	ENSG00000154611	0.01	−0.14	0.01	−0.11	–	0.01

ENSG, Ensembl genes

^a^Information extracted from the Human Protein Atlas (HPA) [24]

^b^Fisher’s combined probability test, adjusted for multiple testing (Benjamini and Hochberg method)

### Plasma profiles associated with VD

**Fig. 4:**
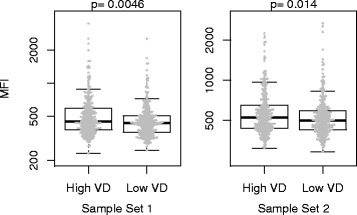
Associations between proteins and volume-based mammographic breast density. Anti-FOXP3 (HPA045943) revealed significantly elevated signal intensities (*p* < 0.05) in women with high absolute volumetric breast density (High VD) compared to women with low absolute volumetric breast density (Low VD). The two samples groups (High VD/Low VD) represent the selection made for the original study design, where the two groups were carefully matched on age and body mass index. Normalised mean fluorescence intensity (MFI) values for women in Sample Set 1 (left) and Sample Set 2 (right) are shown. *P* values were generated using the Wilcoxon signed-rank test

The aforementioned analysis revealed 20 proteins with concordant associations with AD in the two study sets. We also analysed the data in relation to VD in accordance with the initial study design. When comparing women with different VD, we identified significantly elevated levels of forkhead box P3 (FOXP3) using HPA045943 in the high VD group compared to the low VD group (sample set 1, *p* = 0.004; sample set 2, *p* = 0.01; Fig. 4). The anti-FOXP3 antibody, however, was not among the 20 antibodies that were the linearly associated with AD.

### Validation of antibodies

We conducted several experiments to support the indications obtained for the high throughput immunoassays. Acknowledging the challenge to validate antibodies due to their context-dependent and assay-dependent functionality and methods of different sensitivity, we first investigated if paired antibodies raised towards a common protein target would reveal concordant information in the high throughout assay. The data for HPA054101, raised against an internal region of FANCD2 were indeed supported by a second anti-FANCD2 antibody (HPA063742), which was generated against the N-terminal part of the protein. HPA063742 was associated with AD within sample set 1 (*p* = 0.0005) but was not statistically significant in sample set 2 (*p* = 0.09). The two antibodies were correlated, with *rho* = 0.46 (in sample set 1) and *rho* = 0.62 (in sample set 2). Similarly, the association between increasing AD and higher levels of ABCC11 that was observed for HPA031981 was supported by two additional anti- ABCC11 antibodies in sample set 2 (HPA031979; *p* = 0.0004 and HPA031982; *p* = 0.02, respectively). The three anti-ABCC11 antibodies were generated against separate regions of ABCC11.

Next, epitope mapping was conducted on high-density peptide arrays (Additional file 2: Figure S2), which revealed overlapping epitope regions for HPA054101 and four distinct epitope regions for HPA063742**.** None of these epitopes was found to be homologous with abundant plasma proteins, hence supporting on-target recognition of FANCD2 (Additional file 2: Table S6). For HPA064845, raised against ACOX2, four distinct epitope regions were identified. Furthermore, western blot analysis (Additional file 2: Figure S3) revealed a single band within +/− 20% of the expected weight range of ACOX2 (75 kDa). Also, for HPA065387 (TNFRSF10D), which was positively associated with AD in our data, a single band at the expected molecular weight ± 42 kDa was identified. Last, immuno-capture mass spectrometry (IC-MS) was used to assess the selectivity of the highlighted proteins in plasma (Additional file 2: Figure S4). On-target binding was confirmed for six targets, namely ERRF (HPA026676; *z* score = 8.4), RASSF1 (HPA040735; *z* score = 9.1), IL4 (HPA042270; *z* score = 7.5), and ITGB6 (HPA023626; *z* score = 8.1), F11R (HPA061700; *z* score = 3.3) and ABCC11 (HPA031981; *z* score = 11.4). For the latter three, additional proteins were co-enriched, suggesting either multiple off-targets or a complex formation. We suggest a complex has been formed between ITGB6 and LDHA, because of minimal overlap between the HPA023626 antigen region and LDHA (residues CxIxxL). In IC-MS, off-target enrichment was observed for HPA001577 (anti-SHC1; off-target THBS4: *z* score = 4.1) and HPA049377 (anti-PSMA8; off-target LGALS3BP: *z* score = 8.4). Antibodies for FANCD2 (HPA063742) and LIN28B (HPA061745) did not reveal a specific enrichment over the population of commonly identified peptides (*z* score <3). Results from validation experiments are summarised and annotated in Additional file 1: Table S7.

## Discussion

We used antibodies to profile proteins in plasma from healthy women with high and low breast density. Proteins were selected based on their possible linkage to mammographic breast density, cancer development and/or progression or tissue composition and remodelling based on literature review. We identified 20 protein profiles in plasma that were linearly associated with AD in both of the studied sample sets. To our knowledge, this is the first study in which plasma proteins were correlated to AD.

Our study provided indications for eleven candidate proteins for which expression was identified in breast tissue (see Table 2) by analyses of omics data through HPA expression [13] and transcriptome data [25]. Four of these candidates were positively and seven negatively associated with AD. We present a refined description of these proteins and their relation to AD and breast cancer in Additional file 2. There we also explain our perspective on plasma protein associations with age and BMI.

Mammographic density is predominantly associated with higher extracellular matrix (ECM)-rich stromal tissues and epithelial composition, and lower proportion of adipose tissue [17, 26, 27]. High collagen levels in the mouse mammary gland increase tumour formation and invasive behaviour [28], suggesting that dense tissue areas may be tumour promoting. In fact, carcinomas largely arise in the dense region of the breast, supporting the link between tumour formation and mammographic density [6]. Genetic profiles of extra-tumoural stromal microenvironments have identified a so-called “inactive signature”, comprising higher levels of cell adhesion and cell-cell contact genes, associated with higher mammographic density [29, 30]. Collagen-rich stromal tissues are also mechanically stiffer [31, 32], and stiffening of the existing stromal collagen microarchitecture promotes high mammographic density within the breast [33]. Cells sense force and stiffening through mechanoreceptors such as cell-cell junctions and cell-matrix adhesions mediated by integrins, and respond by activating downstream signalling pathways to maintain tissue homeostasis [34, 35]. Consistently, we identified positive association between AD and the epithelial cell-cell adhesion molecule F11R. We also identified negative association with AD and the integrin ITGB6. Elevation of F11R and decrease of ITGB6 in plasma from women with high AD emphasise the complexity of maintaining tissue homeostasis to prevent malignant transformation.

Genetic damage to proliferating cells has been postulated to partake in the increased risk of breast cancer associated with extensive mammographic density [6]. It was recently shown that epithelial cells from high mammographic density tissue have elevated activity in DNA damage signalling, shorter telomeres, and altered DNA damage response compared with epithelial cells from low-density tissues [36]. The authors hypothesise that elevated basal DNA damage in high-density epithelial cells can result in subsequent induction of the desmoplastic-like phenotypes observed in high-density tissues. Therefore, a breast with more DNA-damaged epithelial cells would exhibit more mammographically dense areas, leading to overall high mammographic density. Supporting this hypothesis, we identified two other proteins expressed in breast tissue, namely FANCD2 and RASSF1, which are both related to DNA integrity and were inversely associated with AD. The p53 target gene TNFRSF10D inhibits apoptosis induction and was positively associated with AD in our sample sets. We also observed a negative association with AD and the CASP8 and FADD-like apoptosis regulator CFLAR. Hence, the association of TNFRSF10D and CFLAR plasma levels with high-density tissues could be indicative of mechanisms by which high-density cells avoid apoptosis induced by DNA damage.

The association between endogenous sex hormones and breast cancer risk is widely described; nonetheless, the mechanisms through which sex hormones contribute to mammographic density are complex and incompletely understood. We identified a positive association between the oestrogen receptor (ER)-related nuclear factor ERRF and AD, emphasising the link between oestrogen-mediated signalling and mammographic density.

Both the RAS pathway related protein SHC1, which transmits signalling of cell surface receptors to activate downstream pathways, and the homeobox protein IRX5, involved in cell differentiation and cell cycle regulation, were negatively associated with AD, ss was the acyl-coenzyme A oxidase ACOX2, part of the degradation of long branched fatty acids. AD was also positively associated with the membrane transport-protein ABCC11. Association between AD and proteins involved in cellular proliferation and control of metabolic functions is indicative of the complex dynamic control to maintain an internal steady state in high-density tissue.

Our study has also some limitations. Although we initially selected participants based on volumetric mammographic density, we performed the statistical analyses using the absolute area-based measurement of mammographic density. Current research has led us to believe that area is a better representation of the true dense tissue in the breast and thus the best measurement of mammographic density for analyses of plasma markers of density [23, 37–41]. We also analysed the data in relation to VD in accordance with the initial strategy. When comparing women with different VD, we identified significantly elevated levels of forkhead box P3 (FOXP3) in the high VD compared to the low VD group (Fig. 4). AD and VD are differently associated with age and BMI, which may partly explain this discrepancy (Additional file 2: Table S8). Other limitations are that all exposure data, such as BMI, are self-reported, which may result in some misreporting. However, both exposure data and mammograms were collected at the same time at KARMA study entry. Noticeable is that we used plasma to identify proteins associated with mammographic density. It remains to be ascertained how well blood plasma protein concentrations reflect the protein expression in the breast tissue. Nonetheless, the identified epithelial and stromal cell-specific proteins support protein leakage, shedding or elevated turnaround in breast tissue leading to the detection of these proteins in the circulation. The strengths of our study reside in the large number of samples and the use of two independent sample sets from the KARMA study. This included the centralised collection of mammograms and blood samples, the quantitative assessment of mammographic density by STRATUS, and collection of background information on all participants [15].

The affinity-based assay used in this study provides opportunities for high-throughput screening for novel proteins associated with disease or selected phenotypes. The design allows the combination of different protein assays in one multiplexed approach and it is attractive due to consumption of only minimal volumes of samples. We have taken great care in generating and assessing the data prior to statistical analysis (see Fig. 1) and the candidates presented provide leads for further studies. The method identifies relative protein quantities in plasma and would require the development of assays such as sandwich ELISA for the determination of actual protein concentrations. We have used four different assays to validate the antibodies (see Additional file 2: Figures S4-S6 and Additional file 1: Table S7). This demonstrates the challenge when working with antibodies in exploratory analyses: Depending on the assay sensitivity, sample preparation and target concentration, the performance of the antibody may differ between assays and cannot yet be predicted. Further investigations that preferentially use multiplexed sandwich ELISAs with the shortlisted targets will then allow us to quantify the proteins in abundance to monitor and compare alterations in these in relation to mammographic density in different study sets.

## Conclusion

This study utilised an affinity proteomics approach to explore proteins in plasma associated with mammographic density, aiming at providing molecular insights into mammographic density as a risk factor for breast cancer. We identified a panel of 11 proteins in blood plasma that were associated with mammographic density and also expressed in breast tissue. The candidate proteins have previously been linked to tissue homeostasis, DNA repair and cancer development and/or progression. None, however, have yet been investigated in relation to mammographic density. Our data are indicative of mechanistic processes underlying mammographic breast density and provide insights into the aetiology of breast density as a prominent risk factor for breast cancer. This study further suggests that epithelial-specific and stroma-specific proteins can be found in blood as a consequence of tissue leakage, which would make them key candidates for future individual risk stratification. Each highlighted candidate should be considered during follow-up studies.

## Additional files

Additional file 1: **Tables S1-S2** Complete list of antibodies included in serum bead array (SBA) 1 and 2 (sheet 1 and 2). **Table S7** Summary of antibody validation (sheet 3). (XLSX 71 kb)

Additional file 2: **Tables S3**-**S6**, **S8**, **S9** and **Figures S1**-**S7** Additional material and methods description, results and detailed discussion. (PDF 2026 kb)

## Abbreviations

ABCC11: ATP-binding cassette, sub-family C (CFTR/MRP), member 11
ACOX2: Acyl-CoA oxidase 2, branched chain
AD: Absolute area-based breast density
adj. *p*: Adjusted *p* value
ATR: Ataxia telangiectasia
BMI: Body mass index
BRCA2: Breast cancer 2
CFLAR: CASP8 and FADD-like apoptosis regulator
ECM: Extracellular matrix
ELISA: Enzyme-linked immunosorbent assay
ERRF: ER-related factor (C1orf64, chromosome 1 open reading frame 64)
ERRF: Oestrogen receptor-related nuclear factor
FANCD2: Fanconi anemia complementation group D2
FOXP3: Forkhead box P3
HPA: Human Protein Atlas; IC-MS, Immunocapture mass spectrometry
IHC: Immunohistochemistry
IRX5: Iroquois homeobox 5
ITGB6: Integrin, beta 6
JAM-A: Junction adhesion molecule A (also known as F11R)
KARMA: Karolinska mammography project for risk prediction for breast cancer
PCA: Principal component analysis
PrEST: Protein epitope signature tag
RASSF1: Ras association domain family member 1
RNAseq: RNA sequencing
SBA: Suspension bead array
SHC1: SHC (Src homology 2 domain containing) transforming protein 1
SPNS1: Sphingolipid transporter 1
TGFβ: Transforming growth factor beta 1
TNFRSF10D: Tumour necrosis factor receptor superfamily, member 10d, decoy with truncated death domain
TPP1: Tripeptidyl peptidase 1
VD: Absolute volumetric breast density
