# Supplementary material for: Affinity proteomic profiling of plasma for proteins associated to area-based mammographic breast density
Source: Breast Cancer Res. 2018 Feb 14;20:14. doi: 10.1186/s13058-018-0940-z (PMC5813412; doi:10.1186/s13058-018-0940-z)
Supplement: Supplementary file 2 — Tables S3-S6, S8, S9 and Figures S1-S7 Additional material and methods description, results and detailed discussion. (PDF 2026 kb) [file 13058_2018_940_MOESM2_ESM.pdf]

# Additional information

# Supplementary methods description

### Selection of antibodies

Antibodies were generated within the Human Protein Atlas (HPA) project as described previously [1]. HPA also hosts a portal [www.proteinatlas.org)] which in its current version (v16) provides free access to protein expression analysis with antibodies for ~86% of the human protein-coding genes [2].

For SBA1, the target list was filtered on the availability of antibodies, as evaluated on protein arrays and Western blots. Antibodies were excluded if they 1) showed bands that did not correspond to the predicted size in plasma Western blots or 2) did show cross-reactivity to other proteins during selectivity analysis by protein arrays of 384 PrEST fragments (publically available data) [3]. An additional selection criterion was based on how the antibodies performed in other in-house studies with suspension bead array assays. Here, antibodies were prioritised if – in at least two other sample cohorts – *i)* the median MFI of an antibody was above the median of the negative (rabbit IgG) control bead and *ii)* the biological variance exceeded that of rabbit IgG.

The 393 antibodies included in the second bead array (SBA2) included antibodies selected from immunohistochemistry (IHC) primary data (proteinatlas.org), where the level of staining in breast represented by the cell type with the highest staining score were higher than that of other tissues included in a primary data set (N=19). In addition, a few antibodies with potential relevance for breast cancer (N=12) or other diseases (N=18) according to in-house plasma analysis on suspension bead arrays were included. Both bead arrays (SBA1 and SBA2) included three control bead identities, including one bare bead and bead IDs coupled with normal rabbit IgG (Jacksson ImmunoResearch Laboratories) and anti-human IgG (Dako).

## Details on antibody bead arrays assays

Each antibody was prepared by diluting 1.75 µg in 100 µL of 0.05 M MES buffer (pH 4.5) using liquid handling (EVO150, TECAN). To confirm immobilisation of antibodies, beads were incubated with an R-phycoerythrin-conjugated donkey anti-rabbit IgG antibody (Jackson ImmunoResearch Laboratories).

A liquid handler (Selma, CyBio) was used for all steps involving transferring of liquids. 96 samples from Sample Set 2, originating from one biotinylated 96-well plate, were assayed in duplicate by distributing each duplicate pair across both assay plates. The heating of samples was performed in a water bath (TW8, Julabo) before incubation with the bead array in two 384-well microtiter plates (Greiner BioOne) overnight on a shaking table (Grant). A plate washer (EL406, Biotek) was utilised for all washing steps. Samples were cross-linked with 0.4% paraformaldehyde and detection was mediated through R-phycoerythrin-conjugated streptavidin (Invitrogen) prior parallel measurement of the two assay plates in separate FlexMap3D instruments (Luminex Corp.). All generated data points were based on >50 bead counts per ID and the median fluorescence intensity (MFI) was used to represent the relative amount of target protein binding to the respective bead ID.

## Epitope mapping

The epitope regions of anti-ACOX2 (HPA064845) and anti-FANCD2 antibodies (HPA054101 and HPA063742) were investigated on high-density peptide arrays (Roche NimbleGen), as has previously been described [4]. The in situ synthesized array design included overlapping 16-mer peptides with a 15-residue overlap, which together covered the amino acid sequence of the protein fragment used for antibody generation. The identified epitope regions were searched against the UniProt/Swiss-Prot database [5] using NCBI BLASTp alignment tools. HPA064845, HPA054101, and HPA063742 were incubated on separate arrays at 1 µg/ml and as pools together with 10 additional antibodies against unrelated protein targets. Median fluorescence intensities were extracted from a MS200 microarray scanner (Roche NimbleGen Inc, Madison, WI, USA) and were used to assess the relative binding to the peptides.

## Western blot

Western blot analysis was performed within the Human Protein Atlas [6].

## Immuno-capture mass spectrometry

The selectivity of a subset of candidate antibodies were evaluated by immune-capture mass spectrometry (IC-MS) (**Figure S4**), as has previously been described [7, 8]. In brief, 100 µl plasma (Seralab) from a pool of healthy donors was diluted 1:10, heat-treated and incubated with antibody-coupled magnetic beads (MagPlex, Luminex Corp.) in triplicate. For all assays, beads were washed using a magnetic bead handler (KingFisher Flex, Thermo Scientific), reduced and digested in accordance to previously described protocols. For the generated peptides, the chromatography was conducted using a 50 cm x 75µm ID Easy spray analytical column (PepMap RSLC C18) installed on Ultimate 3000 RSLC nanosystem (Thermo Scientific). Peptide ions were analyzed on a Q-Exactive HF (Thermo) operated in a data dependent mode. Here we investigated antibodies for ACOX2; HPA064845, FANCD2; HPA054101, RASFF1; HPA040735, C1orf64; HPA026676, ITGB6; HPA023626, IL4; HPA042270, LIN28B; HPA063742 and due to availability, antibodies for FANCD2; HPA063742, ABCC11; HPA031981, SHC1; HPA001577 were assessed in duplicate assays. In addition, beads coupled to normal rabbit IgG served as a reference. The obtained data files were processed using the software MaxQuant and searched against a human protein database from Uniprot (http://www.uniprot.org, updated 03/17/2016, Canonical and Isoforms, 20198 hits). Two missing cleavages were allowed. As variable modifications, methionine oxidation and N-term acetylation were selected, while Cysteine carbamidomethylation were selected as a fixed modification. A protein was considered specifically captured by the antibody when one of it's peptides was found to be enriched with z-score > 3 over a population of more than 270 in-house experiments [8].

## Quality assessment of data

The two different bead arrays included 382 and 393 antibodies, with no antibodies or target proteins overlapping between the two arrays. Each bead array was used to

screen 729 (Sample Set 1) and 600 (Sample Set 2) individuals. Data from the four assays were analysed independently (**Figure 1**). In total, 15 individuals were classified as outliers and excluded prior normalization. Data was subsequently filtered to prioritize antibody profiles that we believed were more informative based on 1) median signal intensity above the median intensity level of the negative control bead identity (rabbit IgG), 2) correlation to rabbit IgG with spearman's *rho*<0.4, 3) a correlation between 96 duplicated samples (Sample Set 2) above spearman's *rho* of 0.7, and 4) the ratio between the biological variance for rabbit IgG over the technical variance for a given antibody exceeded the ratio (biological variance/technical variance) for rabbit IgG (**Figure S5**). The technical variance was calculated across pooled replicates and the biological variance across all other samples within an assay (excluding replicates and blank wells). This resulted in exclusion of 138 and 149 antibodies from SBA1 and SBA2 data sets respectively for the proceeding statistical analysis. For all generated data, and after antibody filtering, the median technical variation across all data sets was 6% compared to a biological variation of 28% (**Figure S5**). Normalised signal intensities revealed no differences between the hospitals at which the samples had been collected in any of the four data sets when analysed by PCA (**Figure S6**).

# Additional results

## Results of epitope mapping

Among the epitope regions identified by peptide arrays for the two FANCD2 antibodies were DEFANL, DPKALE, DAFVVD, EGDFPFPVKALY-GLEEYDTQDGIAINLL for HPA054101 and LSSIRLEDL, TLEVISELREKL for HPA063742. Homology searches returned only FANCD2 with significant sequence similarity (E-values <1e-03, **Table S7**). Such homology searches were also performed for HPA064845. The three regions revealing the highest intensity levels were minimal epitopes covering DAILLTDAF, RTAYLDLLR and the less distinct for SGDFLHDAFLSGAQV with E-value of 1e-03, 6e-04 and 2e-09 respectably. This again was a 100% match with the protein sequence identity (**Table S7**).

# Additional discussion

We here present an extension to the discussion presented in the main text, with the intention to complement our perspective about the indicated proteins.

## Description of discovered protein candidates

We found a positive association between AD and the junctional adhesion molecule A (JAM-A), also called platelet factor 11 receptor (F11R) or CD321. This protein is expressed in normal human mammary epithelium where it facilitates cell-to-cell adhesion and prevents migration. The expression of F11R is subsequently down-regulated in metastatic breast cancer tumours [9] by the transforming growth factor beta 1 (TGFβ) to promote breast cancer cell migration and invasion [10]. We also found a negative association with AD and the integrin ITGB6. This protein is expressed at low or very low levels in the normal breast epithelium [11], and is usually increasingly expressed in epithelial cells undergoing tissue remodelling including wound healing and cancer [12]. In breast cancer, ITGB6 activates TGFβ to promote tumour progression [13], which in turn inactivates F11R to facilitate tumour

cell migration and invasion [10]. Although neither F11R nor ITGB6 have been previously investigated in relation to mammographic density, TGFβ has been inversely associated with density and stromal composition, and is suggested to reflect altered stromal-epithelial interactions contributing to the enhanced breast cancer risk of high-density tissues [14, 15]. Elevation of F11R and decrease of ITGB6 in plasma from women with high AD emphasise the complexity of maintaining tissue homeostasis to prevent malignant transformation.

FANCD2 act as components of the DNA repair system and co-localises with BRCA2 amongst other proteins in response to DNA damage [16, 17]. It is also involved during the S-phase of the normal cell cycle [18], and in replicative stress [19]. Thus, disruption or mutation of the nuclear FANCD2 core complex impairs DNA repair processes [20, 21]. Germline disruption of the FANCD2 gene results in ovarian and epithelial breast cancers in mouse model studies [22, 23]. Furthermore, altered FANCD2 has been associated with sporadic breast cancer risk [24], shorter time to recurrence [25] and poorer breast cancer survival [26]. There is currently great interest in using FANCD2 foci formation as a functional biomarker to predict the sensitivity of cancer cells to DNA crosslinking chemotherapy [27].

The RASSF1 gene is frequently epigenetically silenced by hypermethylation in cancer [28-30], with hypermethylation of the transcript variant RASSF1A in 60–77% of breast cancers [31, 32]. RASSF1 is a tumour suppressor gene that forms part of an ATR mediated response to replication stress, and perturbation of the signalling axis leads to genomic defects [33]. It also influences the G1-S cell cycle checkpoint by regulating the level of cyclin D1 protein [34]. Interestingly, Lewis and colleagues have shown that epigenetic silencing of RASSF1 by promotor methylation occurred more frequently in unaffected women at high-risk for breast cancer as defined by the Gail model than in low/intermediate risk women [31]. The authors suggest that silencing of RASSF1 represent one mechanism for over riding cell cycle control under conditions of increased cell cycle pressure, and which could be exploited for tissue-based individualised breast cancer risk stratification. Supported by our findings,

serum based methylation analysis of RASSF1 has been proposed as a prognostic tool for breast cancer [35].

The p53 target gene TNFRSF10D inhibits apoptosis induction [36] and was positively associated with AD in our sample sets. Overexpression of TNFRSF10D in cancer cells protected cells against apoptosis induced by DNA-damaging agents [36]. It has also been shown that reduced TNFRSF10D expression appears to be associated with reduced breast cancer risk [37]. Hence, increased plasma levels point at induced expression of TNFRSF10D in high-density tissues, which could be indicative for mechanisms by which high-density cells avoid apoptosis induced by DNA damage.

We found a positive association between the ER-related nuclear factor ERRF and AD in both study sets. Up-regulated expression of ERRF is associated with oestrogen and/or progesterone receptor positive breast cancer [38], and oestrogen-mediated signalling supposedly regulates ERRF to promote breast cancer cell growth [39]. We have previously shown that stromal expression of ER is associated with greater stromal proportion of breast tissue in healthy women, whereas epithelial ER is not associated to neither epithelial nor stromal content [40]. Sun and colleagues further demonstrated a positive association between mammographic density and ER gene expression in non-neoplastic breast tissues [14]. Although the association between endogenous sex hormones and breast cancer risk is widely described, the mechanisms through which sex hormones contribute to mammographic density are complex and incompletely understood. To our current knowledge, this is the first study to show an association between ERRF and mammographic density.

**Association to BMI:**

We also investigated the relation between the protein profiles in plasma to age and BMI. Particularly for age, we observed several strong associations ($p<0.001$). Many of the shortlisted proteins were among those associated to age and BMI (Table S3 and Table S4, respectively), hence we included these co-variates in the statistical models for the analysis of AD. This also confirms previous studies [41, 42] and demands to carefully assess the relation of a protein profile in plasma with other clinical data. For

the top association to age was SPNS1, which belong to the Spinster family of facilitator proteins. SPNS1 is suggested to partake in regulation of autolysosome biogenesis and developmental senescence [43]. To BMI, it was TPP1 that revealed the strongest association. Interestingly, TPP1 is a metabolic enzyme involved in adipogenesis [44]. Belonging to the same family of proteins, TPP2 promotes fat formation in a conserved fashion [45]. To the best of out knowledge, this is the first study to show an association of SPNS1 and TPP1 with age and BMI, respectively.

# Additional references

1. Nilsson, P., et al., *Towards a human proteome atlas: high-throughput generation of mono-specific antibodies for tissue profiling.* Proteomics, 2005. **5**(17): p. 4327-37.
2. Uhlen, M., et al., *Towards a knowledge-based Human Protein Atlas.* Nat Biotechnol, 2010. **28**(12): p. 1248-50.
3. Sjoberg, R., et al., *Exploration of high-density protein microarrays for antibody validation and autoimmunity profiling.* N Biotechnol, 2016. **33**(5 Pt A): p. 582-92.
4. Forsstrom, B., et al., *Proteome-wide epitope mapping of antibodies using ultra-dense peptide arrays.* Mol Cell Proteomics, 2014. **13**(6): p. 1585-97.
5. *The Universal Protein Resource (UniProt).* [Internet Database] 2017 03/17/2016 [cited 2016 03/17/2016]; Available from: http://www.uniprot.org/.
6. Algenas, C., et al., *Antibody performance in western blot applications is context-dependent.* Biotechnol J, 2014. **9**(3): p. 435-45.
7. Remnestal, J., et al., *CSF profiling of the human brain enriched proteome reveals associations of neuromodulin and neurogranin to Alzheimer's disease.* Proteomics Clin Appl, 2016. **10**(12): p. 1242-1253.
8. Fredolini, C., et al., *Mass spectrometry based qualification of antibodies for plasma proteomics.*, S.o.B. Science for Life Laboratory, KTH - Royal Institute of Technology, Editor. 2017: bioRxiv beta. Unpublished results.
9. Naik, M.U., et al., *Attenuation of junctional adhesion molecule-A is a contributing factor for breast cancer cell invasion.* Cancer Res, 2008. **68**(7): p. 2194-203.
10. Wang, Y. and W.Y. Lui, *Transforming growth factor-beta1 attenuates junctional adhesion molecule-A and contributes to breast cancer cell invasion.* Eur J Cancer, 2012. **48**(18): p. 3475-87.
11. Breuss, J.M., et al., *Restricted distribution of integrin beta 6 mRNA in primate epithelial tissues.* J Histochem Cytochem, 1993. **41**(10): p. 1521-7.
12. Breuss, J.M., et al., *Expression of the beta 6 integrin subunit in development, neoplasia and tissue repair suggests a role in epithelial remodeling.* J Cell Sci, 1995. **108 ( Pt 6)**: p. 2241-51.
13. Allen, M.D., et al., *Altered microenvironment promotes progression of preinvasive breast cancer: myoepithelial expression of alphavbeta6 integrin in DCIS identifies high-risk patients and predicts recurrence.* Clin Cancer Res, 2014. **20**(2): p. 344-57.
14. Sun, X., et al., *Relationship of mammographic density and gene expression: analysis of normal breast tissue surrounding breast cancer.* Clin Cancer Res, 2013. **19**(18): p. 4972-82.
15. Yang, W.T., et al., *Decreased TGFbeta signaling and increased COX2 expression in high risk women with increased mammographic breast density.* Breast Cancer Res Treat, 2010. **119**(2): p. 305-14.
16. Kim, H. and A.D. D'Andrea, *Regulation of DNA cross-link repair by the Fanconi anemia/BRCA pathway.* Genes Dev, 2012. **26**(13): p. 1393-408.

17. Hussain, S., et al., *Direct interaction of FANCD2 with BRCA2 in DNA damage response pathways.* Hum Mol Genet, 2004. **13**(12): p. 1241-8.
18. Taniguchi, T., et al., *S-phase-specific interaction of the Fanconi anemia protein, FANCD2, with BRCA1 and RAD51.* Blood, 2002. **100**(7): p. 2414-20.
19. Howlett, N.G., et al., *The Fanconi anemia pathway is required for the DNA replication stress response and for the regulation of common fragile site stability.* Hum Mol Genet, 2005. **14**(5): p. 693-701.
20. Nakanishi, K., et al., *Human Fanconi anemia monoubiquitination pathway promotes homologous DNA repair.* Proc Natl Acad Sci U S A, 2005. **102**(4): p. 1110-5.
21. Garcia-Higuera, I., et al., *Interaction of the Fanconi anemia proteins and BRCA1 in a common pathway.* Mol Cell, 2001. **7**(2): p. 249-62.
22. Houghtaling, S., et al., *Heterozygosity for p53 (Trp53+/-) accelerates epithelial tumor formation in fanconi anemia complementation group D2 (Fancd2) knockout mice.* Cancer Res, 2005. **65**(1): p. 85-91.
23. Houghtaling, S., et al., *Epithelial cancer in Fanconi anemia complementation group D2 (Fancd2) knockout mice.* Genes Dev, 2003. **17**(16): p. 2021-35.
24. Barroso, E., et al., *FANCD2 associated with sporadic breast cancer risk.* Carcinogenesis, 2006. **27**(9): p. 1930-7.
25. Alexander, B.M., et al., *DNA repair protein biomarkers associated with time to recurrence in triple-negative breast cancer.* Clin Cancer Res, 2010. **16**(23): p. 5796-804.
26. Rudland, P.S., et al., *Significance of the Fanconi anemia FANCD2 protein in sporadic and metastatic human breast cancer.* Am J Pathol, 2010. **176**(6): p. 2935-47.
27. Kennedy, R.D. and A.D. D'Andrea, *DNA repair pathways in clinical practice: lessons from pediatric cancer susceptibility syndromes.* J Clin Oncol, 2006. **24**(23): p. 3799-808.
28. Burbee, D.G., et al., *Epigenetic inactivation of RASSF1A in lung and breast cancers and malignant phenotype suppression.* J Natl Cancer Inst, 2001. **93**(9): p. 691-9.
29. Jiang, Y., et al., *The prognostic role of RASSF1A promoter methylation in breast cancer: a meta-analysis of published data.* PLoS One, 2012. **7**(5): p. e36780.
30. Wang, J., et al., *The prognostic value of RASSF1A promoter hypermethylation in non-small cell lung carcinoma: a systematic review and meta-analysis.* Carcinogenesis, 2011. **32**(3): p. 411-6.
31. Lewis, C.M., et al., *Promoter hypermethylation in benign breast epithelium in relation to predicted breast cancer risk.* Clin Cancer Res, 2005. **11**(1): p. 166-72.
32. Vincent-Salomon, A., et al., *X inactive-specific transcript RNA coating and genetic instability of the X chromosome in BRCA1 breast tumors.* Cancer Res, 2007. **67**(11): p. 5134-40.
33. Pefani, D.E., et al., *RASSF1A-LATS1 signalling stabilizes replication forks by restricting CDK2-mediated phosphorylation of BRCA2.* Nat Cell Biol, 2014. **16**(10): p. 962-71, 1-8.

34. Shivakumar, L., et al., *The RASSF1A tumor suppressor blocks cell cycle progression and inhibits cyclin D1 accumulation.* Mol Cell Biol, 2002. **22**(12): p. 4309-18.
35. Muller, H.M., et al., *DNA methylation in serum of breast cancer patients: an independent prognostic marker.* Cancer Res, 2003. **63**(22): p. 7641-5.
36. Liu, X., et al., *Decoy receptor 2 (DcR2) is a p53 target gene and regulates chemosensitivity.* Cancer Res, 2005. **65**(20): p. 9169-75.
37. Martinez-Ferrandis, J.I., et al., *Polymorphisms in TRAIL receptor genes and risk of breast cancer in Spanish women.* Cancer Biomark, 2007. **3**(2): p. 89-93.
38. Su, D., et al., *Role of ERRF, a novel ER-related nuclear factor, in the growth control of ER-positive human breast cancer cells.* Am J Pathol, 2012. **180**(3): p. 1189-201.
39. Luo, A. and X. Zhang, *ERRF is essential for Estrogen-Estrogen Receptor alpha signaling pathway in ER positive breast cancer cells.* Biochem Biophys Res Commun, 2016. **474**(2): p. 400-5.
40. Gabrielson, M., et al., *Amount of stroma is associated with mammographic density and stromal expression of oestrogen receptor in normal breast tissues.* Breast Cancer Res Treat, 2016.
41. Bartow, S.A., et al., *Breast mammographic pattern: a concatenation of confounding and breast cancer risk factors.* Am J Epidemiol, 1995. **142**(8): p. 813-9.
42. Ignjatovic, V., et al., *Age-related differences in plasma proteins: how plasma proteins change from neonates to adults.* PLoS One, 2011. **6**(2): p. e17213.
43. Sasaki, T., et al., *Autolysosome biogenesis and developmental senescence are regulated by both Spns1 and v-ATPase.* Autophagy, 2017. **13**(2): p. 386-403.
44. Ye, F., et al., *Comparative proteome analysis of 3T3-L1 adipocyte differentiation using iTRAQ-coupled 2D LC-MS/MS.* J Cell Biochem, 2011. **112**(10): p. 3002-14.
45. McKay, R.M., et al., *Tripeptidyl peptidase II promotes fat formation in a conserved fashion.* EMBO Rep, 2007. **8**(12): p. 1183-9.

# Additional tables

## Table S1. Complete list of antibodies included in serum bead array (SBA) 1.

All antibodies included in SBA1. Controls bead IDs (rIgG, anti-hIgG, anti-albumin, bare bead) are not listed.

See Additional file 2; Bystrom_revised_additional_file2_BCR.xlsx. Sheet 1.

## Table S2. Complete list of antibodies included in serum bead array (SBA) 2.

All antibodies included in SBA2. Controls bead IDs (rIgG, anti-hIgG, bare bead) are not listed.

See Additional file 2; Bystrom_revised_additional_file2_BCR.xlsx. Sheet 2.

## Table S3: Associations of plasma protein profiles with age in both sample sets.

Both age and BMI influence AD but were not associated with one another in the studied sample sets ($p>0.1$). For age, 11 plasma profiles reached association levels of $p<10^{-10}$ with concordant trends in both sample sets.

| Gene | HPA | ENSG | Age | | | | | BMI | | | | |
|---|---|---|---|---|---|---|---|---|---|---|---|---|
| | | | Sample Set 1, N=729 | | Sample Set 2, N=600 | | Trend | Sample Set 1, N=729 | | Sample Set 2, N=600 | | Trend |
| | | | p-value | Effect size | p-value | Effect size | | p-value | Effect size | p-value | Effect size | |
| ACOX2 | HPA064845 | ENSG00000168306 | 5.47E-20 | 9.51E-03 | 6.84E-13 | 8.49E-03 | + | 4.88E-09 | 1.94E-02 | 2.23E-13 | 3.06E-02 | + |
| AMBN | HPA050143 | ENSG00000178522 | 3.67E-21 | -1.77E-02 | 7.08E-17 | -1.46E-02 | - | 1.19E-02 | -1.52E-02 | 1.21E-01 | -9.91E-03 | - |
| EED | HPA061140 | ENSG00000074266 | 1.20E-18 | -5.16E-03 | 3.85E-16 | -4.05E-03 | - | 1.62E-04 | -7.10E-03 | 5.00E-05 | -7.32E-03 | - |
| ITGB6 | HPA023626 | ENSG00000115221 | 4.43E-19 | 7.39E-03 | 2.83E-11 | 5.45E-03 | + | 1.07E-08 | 1.52E-02 | 7.37E-12 | 1.99E-02 | + |
| MLH1 | HPA052707 | ENSG00000076242 | 7.86E-16 | -8.03E-03 | 5.52E-17 | -8.86E-03 | - | 3.00E-01 | -3.32E-03 | 2.02E-01 | -4.91E-03 | - |
| PSMA8 | HPA049377 | ENSG00000154611 | 2.14E-13 | 9.53E-03 | 1.25E-13 | 1.01E-02 | + | 1.23E-04 | 1.59E-02 | 1.14E-11 | 3.29E-02 | + |
| PTGR1 | HPA036725 | ENSG00000106853 | 2.96E-12 | -3.08E-03 | 7.82E-15 | -3.76E-03 | - | 5.27E-03 | -3.92E-03 | 1.66E-02 | -4.19E-03 | - |
| SHC1 | HPA001577 | ENSG00000160691 | 4.08E-11 | 6.43E-03 | 9.06E-12 | 1.06E-02 | + | 1.06E-04 | 1.20E-02 | 3.10E-16 | 4.46E-02 | + |
| SPNS1 | HPA041995 | ENSG00000169682 | 5.80E-35 | 1.01E-02 | 2.07E-26 | 8.03E-03 | + | 1.94E-03 | 8.41E-03 | 6.30E-03 | 7.67E-03 | + |
| TGFA | HPA042297 | ENSG00000163235 | 1.32E-14 | -5.04E-03 | 3.75E-11 | -5.83E-03 | - | 4.39E-05 | -8.52E-03 | 1.17E-01 | -4.98E-03 | - |
| TMEM86A | HPA057119 | ENSG00000151117 | 2.14E-16 | -2.40E-02 | 3.99E-18 | -2.31E-02 | - | 7.18E-02 | -1.69E-02 | 9.08E-03 | -2.55E-02 | - |

## Table S4: Associations of plasma protein profiles with BMI in both sample sets.

Both age and BMI influence AD but were not associated with one another in the studied sample sets (p>0.1). For BMI, 10 plasma profiles reached association levels of $p<10^{-5}$ with concordant trends in both sample sets.

| Gene | HPA | ENSG | BMI | | | | | Age | | | | |
|---|---|---|---|---|---|---|---|---|---|---|---|---|
| | | | Sample Set 1, N=729 | | Sample Set 2, N=600 | | Trend | Sample Set 1, N=729 | | Sample Set 2, N=600 | | Trend |
| | | | p-value | Effect size | p-value | Effect size | | p-value | Effect size | p-value | Effect size | |
| ACOX2 | HPA064845 | ENSG00000168306 | 4.88E-09 | 1.94E-02 | 2.23E-13 | 3.06E-02 | + | 5.47E-20 | 9.51E-03 | 6.84E-13 | 8.49E-03 | + |
| CASP2 | HPA042253 | ENSG00000106144 | 3.85E-08 | 1.90E-02 | 1.20E-13 | 3.61E-02 | + | 6.03E-07 | 5.50E-03 | 3.27E-10 | 8.71E-03 | + |
| CFH | HPA053326 | ENSG00000000971 | 4.59E-12 | 9.91E-03 | 3.07E-06 | 6.12E-03 | + | 5.53E-08 | 3.42E-03 | 5.53E-08 | 2.00E-03 | + |
| ENG | HPA011143 | ENSG00000106991 | 4.10E-09 | -1.18E-02 | 2.30E-06 | -7.68E-03 | - | 1.72E-02 | -1.53E-03 | 1.72E-02 | -1.10E-03 | - |
| IL4 | HPA042270 | ENSG00000113520 | 2.05E-08 | 1.07E-02 | 7.45E-09 | 1.49E-02 | + | 6.09E-12 | 3.87E-03 | 6.09E-12 | 4.98E-03 | + |
| ITGB6 | HPA023626 | ENSG00000115221 | 1.07E-08 | 1.52E-02 | 7.37E-12 | 1.99E-02 | + | 2.83E-11 | 7.39E-03 | 2.83E-11 | 5.45E-03 | + |
| LAMB2 | HPA001859 | ENSG00000172037 | 3.15E-13 | 1.63E-02 | 5.91E-11 | 1.15E-02 | + | 1.80E-07 | 4.12E-03 | 1.80E-07 | 2.59E-03 | + |
| SERPINB12 | HPA058583 | ENSG00000166634 | 1.24E-08 | 1.14E-02 | 1.59E-06 | 9.13E-03 | + | 1.28E-06 | 4.65E-03 | 1.28E-06 | 2.59E-03 | + |
| SUCLA2 | HPA039435 | ENSG00000136143 | 1.28E-11 | 1.70E-02 | 2.32E-15 | 2.62E-02 | + | 1.36E-10 | 5.65E-03 | 1.36E-10 | 6.06E-03 | + |
| TPP1 | HPA037710 | ENSG00000166340 | 2.30E-20 | 2.16E-02 | 1.12E-12 | 1.99E-02 | + | 1.51E-01 | -1.78E-03 | 1.51E-01 | -1.16E-03 | - |

## Table S5: Annotated gene expression of candidate proteins expressed in breast tissue.

Eleven proteins were associated to absolute area-based mammographic breast density (AD) (p<5e-02), showing RNA expression, transcripts or protein expression in breast tissue according to publically available data sets (Gtex and HPA data sets, available at [proteinatlas.org]).

| Gene | HPA | Gtex portal RPKM* | GTEx portal RNA Class | HPA portal TPM** | HPA portal RNA Class | Protein expression IHC |
|---|---|---|---|---|---|---|
| ABCC11 | HPA031981 | 1.4 | Group enriched (testis, **breast**, liver, prostate) | 63.2 | Tissue enriched (**breast**) | medium (3) |
| ACOX2 | HPA064845 | 7.2 | Expressed in all | 4.3 | Liver enhanced | low (3) |
| CFLAR | HPA050009 | 14 | Expressed in all | 62.7 | Expressed in all | medium (3) |
| ERRF | HPA026676 | 2.9 | Tissue enhanced (caudate, **breast**) | 7 | Tissue enhanced (**breast**) | NA |
| F11R | HPA061700 | 0 | Not detected | 32.3 | Expressed in all | low (2) |
| FANCD2 | HPA054101 | 1 | Tissue enhanced (testis) | 4.1 | Mixed | not detected (2) |
| IRX5 | HPA047130 | 12.3 | Tissue enhanced (**breast**, skin) | 18.6 | Tissue enhanced (**breast**, skin, lung) | NA |
| ITGB6 | HPA023626 | 3.6 | Tissue enhanced (testis, kidney) | 24.5 | Tissue enhanced (lung) | not detected (3) |
| RASSF1 | HPA040735 | 24.5 | Expressed in all | 22.7 | Expressed in all | medium (1) |
| SHC1 | HPA001577 | 61.7 | Expressed in all | 63.7 | Expressed in all | medium (3) |
| TNFRSF10D | HPA065387 | 4 | Expressed in all | 3.1 | Mixed | NA |

*Mean levels of RNA transcripts in breast (214 samples).

**Mean RNA expression in breast (4 samples). Data publically available at [proteinatlas.org].

## Table S6. Epitope homology searches of ACOX2 and FANCD2.

The table shows the highest and the second highest scoring alignments from homology searches (BLASTp). Stars (*) indicate alignments to targeted proteins.

| Gene Antibody | Query epitope region | Gene name | E value | Coverage [%] | Sequence identity |
|---|---|---|---|---|---|
| ACOX2 HPA064845 | DAILLTDAF | Acyl-coenzyme A oxidase 2* | 0.001 | 100 | 100 |
| | | Acetyl-CoA acetyltransferase | 0.69 | 100 | 67 |
| | RTAYLDLLR | Peroxisomal acyl-coenzyme A oxidase 2* | 6.0E-04 | 100 | 100 |
| | | integral membrane protein 2A | 0.17 | 77 | 100 |
| | SGDFLHDAFLSGAQV | Peroxisomal acyl-coenzyme A oxidase 2* | 2.0E-09 | 100 | 100 |
| | | Oral-facial-digital syndrome 1 protein | 6.2 | 60 | 67 |
| | | Spectrin alpha chain, non-erythrocytic 1 | 6.2 | 60 | 67 |
| | | Dynein heavy chain 2, axonemal | 6.2 | 73 | 70 |
| FANCD2 HPA063742 | LSSIRLEDL | Fanconi anemia group D2 protein* | 0.002 | 100 | 100 |
| | | Transmembrane 9 superfamily member 4 | 0.17 | 77 | 100 |
| | TLEVISELREKL | Fanconi anemia group D2 protein* | 1.0E-06 | 100 | 100 |
| | | RING finger protein 39 | 0.16 | 83 | 83 |
| FANCD2 HPA054101 | DEFANL | G-protein coupled receptor 98 | 0.67 | 100 | 100 |
| | | Polycystic kidney disease protein 1-like 1 | 0.67 | 100 | 100 |
| | | Fanconi anemia group D2 protein* | 0.68 | 100 | 100 |
| | DPKALE | Fanconi anemia group D2 protein | 1.4 | 100 | 100 |
| | | Catenin alpha-2 | 11 | 100 | 83 |
| | | Catenin alpha-1 | 11 | 100 | 83 |
| | | Retinoid-inducible serine carboxypeptidase | 11 | 100 | 83 |
| | DAFVVD | Fanconi anemia group D2 protein* | 0.96 | 100 | 100 |
| | | Fibrous sheath-interacting protein 2 | 16 | 83 | 100 |
| | | Mucin-5AC | 16 | 83 | 100 |
| | | von Willebrand factor | 16 | 83 | 100 |
| | EGDFPFPVKALYGLEEYDTQDGIAINLL | Fanconi anemia group D2 protein* | 4.0E-24 | 100 | 100 |
| | | Sulfotransferase family cytosolic 2B member 1 | 0.26 | 60 | 55 |
| | EGDFPFPV | Fanconi anemia group D2 protein* | 3.0E-03 | 100 | 100 |
| | | MTSS1-like protein | 1.1 | 87 | 86 |

## Table S7. Summary of antibody validation.

See Additional file 2; Bystrom_revised_additional_file2_BCR.xlsx. Sheet 3.

**Table S8. Spearman's correlations between age or BMI and absolute density measures in the four data sets.**

| | Age and VD | | Age and AD | | BMI and VD | | BMI and AD | |
|---|---|---|---|---|---|---|---|---|
| Sample set | *rho* | p-value | *rho* | p-value | *rho* | p-value | *rho* | p-value |
| Sample Set 1: SBA1 | -0,18 | <0.001 | -0,35 | <0.001 | 0,15 | <0.001 | -0,28 | <0.001 |
| Sample Set 1: SBA2 | -0,16 | <0.001 | -0,35 | <0.001 | 0,13 | <0.001 | -0,28 | <0.001 |
| Sample Set 2: SBA1 | -0,16 | <0.001 | -0,27 | <0.001 | 0,21 | <0.001 | -0,22 | <0.001 |
| Sample Set 2: SBA2 | -0,16 | <0.001 | -0,27 | <0.001 | 0,21 | <0.001 | -0,22 | <0.001 |
| Mean, all Sample Sets | **-0,17** | | **-0,31** | | **0,18** | | **-0,25** | |

Abbreviations: AD, absolute area-based mammographic breast density; BMI, body mass index; SBA, suspension bead array; VD, absolute volumetric breast density.

# Additional figures

**Figure S1. Correlation of absolute area-based mammographic breast density to BMI and age.**

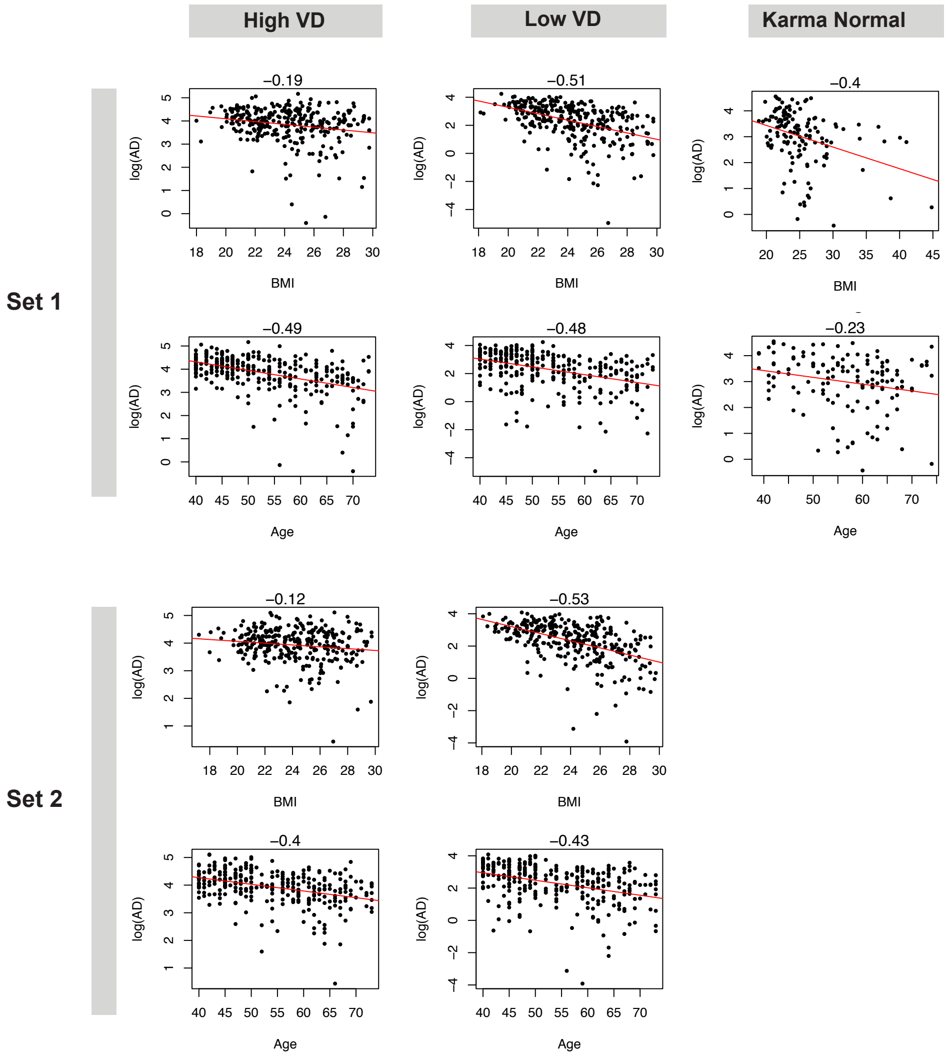


Correlation between absolute area-based mammographic breast density (AD) and BMI or age are illustrated for the different sample groups within each sample set of samples selected for the initial study design (Sample set 1: High VD, Low VD and KarmaNormal; Sample Set 2: High VD and Low VD). The values above each plot represent Spearman's correlation coefficients (*rho*). AD shows a stronger negative correlation to BMI in the Low VD group (Sample Set 1; *rho*=-0.52, Sample Set 2; *rho*=-0.53) compared to the High VD group (Sample Set 1; *rho*=-0.19, Sample Set 2; *rho*=-0.12).

## Figure S2. Epitope mapping anti-ACOX2 and anti-FANCD2

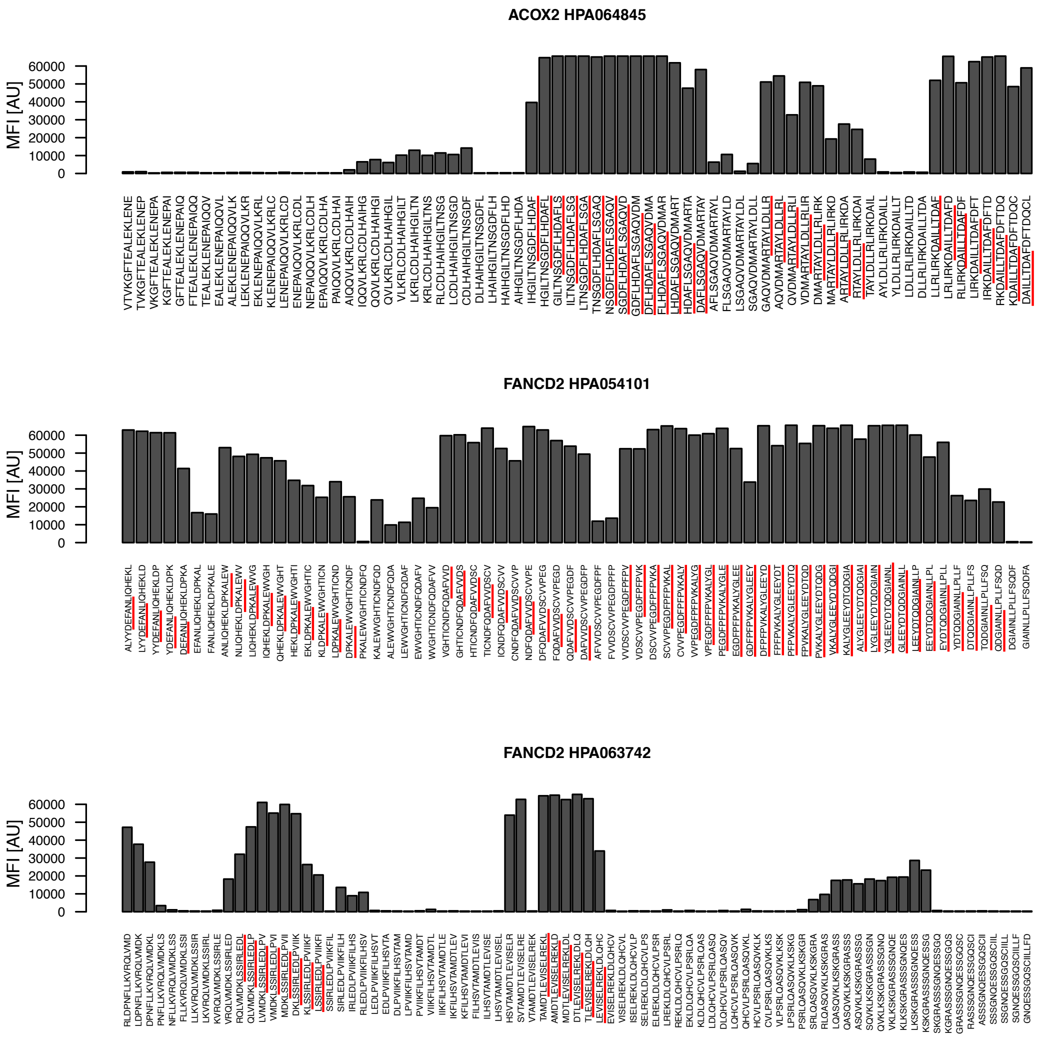


High-density peptide arrays were used for epitope mapping of anti-ACOX2 (HPA064845) and anti-FANCD2 antibodies (HPA054101 and HPA063742). 16-mer peptides with 15 residue overlaps (x-axis) covered the antigen regions that were used for immunization and purification of antibodies. The highest signal intensity regions are indicated in red and were used for homology searches presented in **Table S7**.

## Figure S3. Western blot ACOX2 and TNFRSF10D.

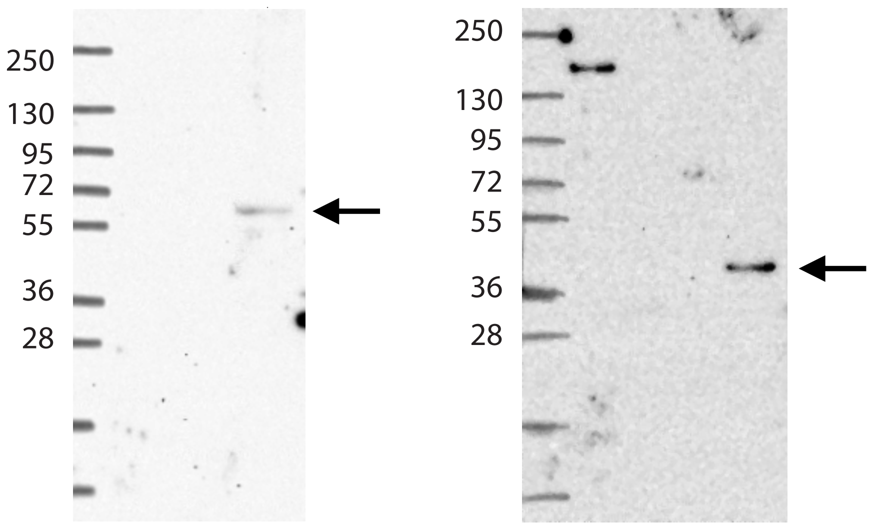


ACOX2 HPA064845
ENST00000459701: 75.1 kDa
ENST00000302819: 76.8 kDa

HPA065387_TNFRSF10
ENST00000312584: 41.8 kDa

Western blot analysis of anti-ACOX2 and anti-TNFRSF10 antibodies showed a single band in depleted human plasma (rightmost lane).

**Figure S4. Immune-capture mass spectrometry validation of protein candidates.**

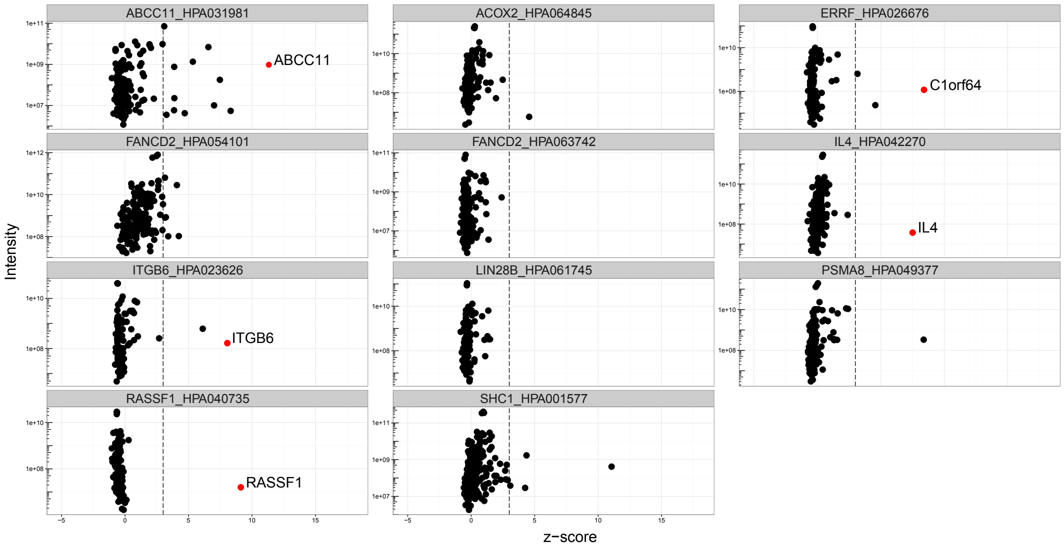


Protein candidates were validated by immune-capture mass spectrometry (IC-MS). As exemplified for a selected set of candidates, z-scores (x-axes) are plotted against protein intensity (y-axes) for each protein identified by IC-MS performed with (ABCC11; HPA031981, ACOX2; HPA064845, ERRF; HPA026676, FANCD2; HPA054101, FANCD2; HPA063742, IL4; HPA042270, ITGB6; HPA023626, LIN28B; HPA063742, PSMA8; HPA049377, RASFF1; HPA040735 and SHC1; HPA001577).

# Figure S5. Quality assessment of the data.

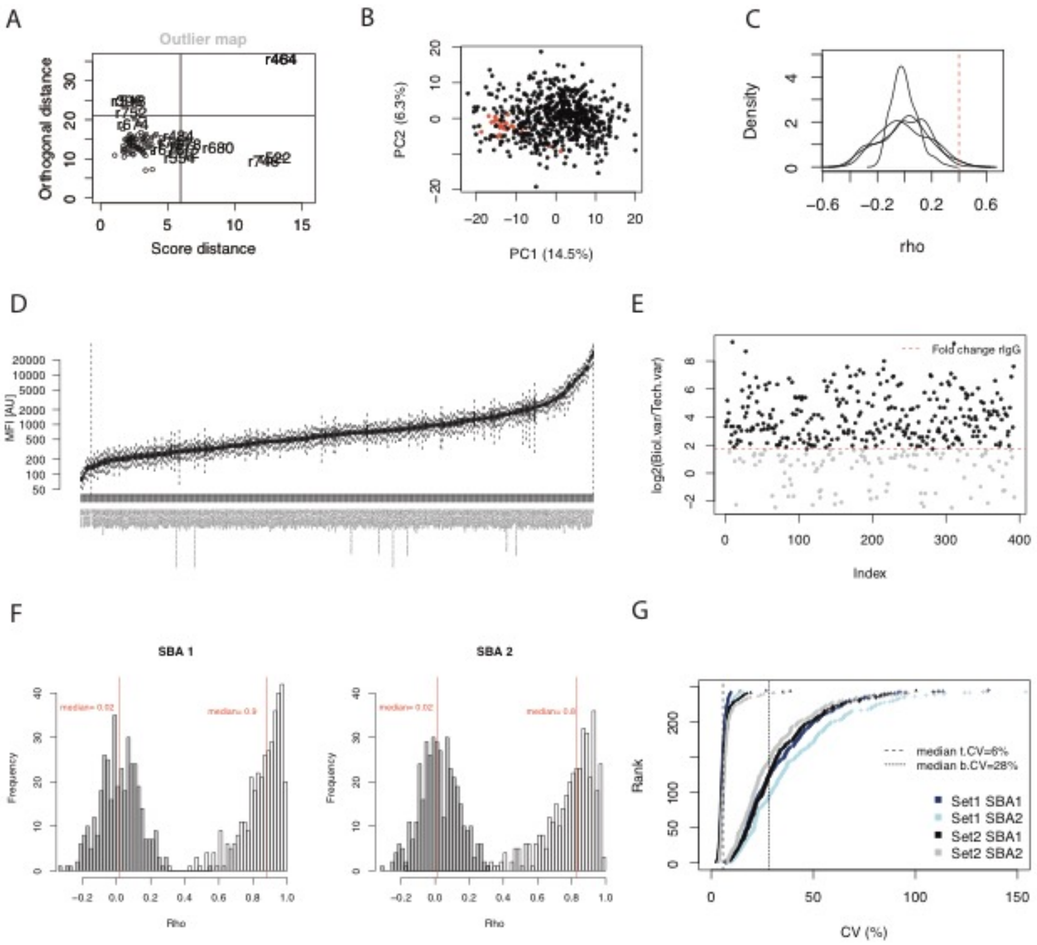


Technical evaluation of data generated from antibody suspension bead arrays. **(A)** Unprocessed data was subjected to robust principal component analysis (PCA) to identify potential sample outliers. As exemplified in the plot showing data from one 96-well plate, sample ID 464 was classified as an outlier and subsequently removed prior data normalisation. **(B)** PCA shows the clustering of 16 replicates of one pooled sample (red) among all other biological samples measured within one SBA after data normalisation and outlier removal. **(C-E)**: Normalised data was filtered for antibody profiles based on technical assay performance: **(C)** The density plots (one line per data set) show the distribution of Spearman's correlation coefficients (*rho*) between antibody profiles and rabbit IgG (rIgG). Antibodies with a correlation to rIgG of <0.4 (dashed line) were considered for statistical analysis. **(D)** The boxplots show the distribution of normalised MFI (y-axis) of all antibodies (x-axis), exemplified by data

from Sample Set 2 SBA2. Antibodies with median signals in between the median signal intensity of rIgG and anti-human IgG (dashed lines) were selected for further analysis. **(E)** The biological variance over technical variance for each antibody, exemplified by data from Sample Set 2 SBA2. Antibodies with a ratio below the ratio of rIgG (grey dots) were filtered away. **(F)** The histograms show the distribution of Spearman's correlation coefficients between antibody profiles that were generated from 96 duplicated measurements in Sample Set 2 (SBA1; median *rho* = 0.8, SBA2; median *rho*=0.9), alongside correlation coefficients generated from a random set of 96 samples (SBA1; median *rho* = 0.8, SBA2; median *rho*=0.9). Only antibodies with *rho* > 0.7 were considered for statistical analysis. **(G)** After antibody filtering, the technical CV (t.CV) were computed. The plot illustrates the ranked t.CV and biological CV (b.CV) of each antibody (color-coded by data set).

# Figure S6. Principal component analysis of site of sample collection.

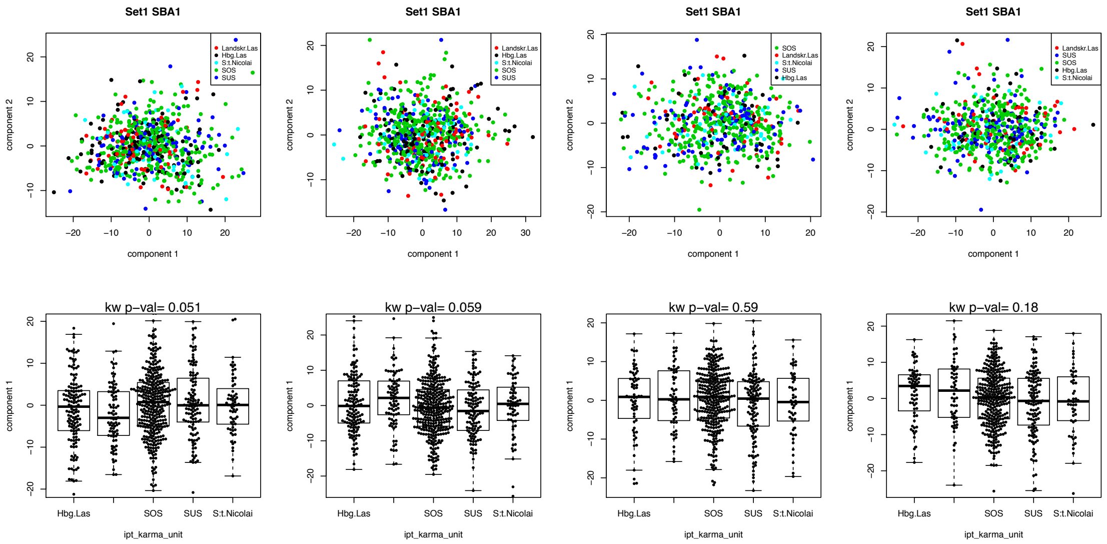


Samples were collected at five different hospitals (denoted Hbg.Las, Landskr.Las, SOS, SUS, and S:t.Nicolai). As illustrated for each column and data set (Sample Set1/SBA1; Sample Set1/SBA2; Sample Set2/SBA1; Sample Set2/SBA2), principal component analysis revealed no effects related to site of sample collection. Upper panel: The first two components are plotted for log-transformed and feature scaled data. Samples are colour coded by the site of sample collection. Lower panel: Boxplots illustrate the distribution of the first principal component across the five different hospitals, with a p-value from Kruskal-Wallis tests indicated above each plot.
